# DVH Prediction for VMAT in NPC with GRU-RNN: An Improved Method by Considering Biological Effects

**DOI:** 10.1155/2021/2043830

**Published:** 2021-01-19

**Authors:** Yongdong Zhuang, Yaoqin Xie, Luhua Wang, Shaomin Huang, Li-Xin Chen, Yuenan Wang

**Affiliations:** ^1^Department of Radiation Oncology, National Cancer Center/National Clinical Research Center for Cancer/Cancer Hospital & Shenzhen Hospital, Chinese Academy of Medical Sciences and Peking Union Medical College, Shenzhen 518116, China; ^2^Institute of Biomedical and Health Engineering, Shenzhen Institutes of Advanced Technology, Chinese Academy of Sciences, Shenzhen 518055, China; ^3^Department of Radiation Oncology, National Cancer Center/National Clinical Research Center for Cancer/Cancer Hospital, Chinese Academy of Medical Sciences and Peking Union Medical College, Beijing 100021, China; ^4^Department of Radiation Oncology, Sun Yat-sen University Cancer Center, State Key Laboratory of Oncology in South China, Guangzhou 510060, China; ^5^Department of Radiation Oncology, Peking University Shenzhen Hospital, Shenzhen 518036, China

## Abstract

**Purpose:**

A recurrent neural network (RNN) and its variants such as gated recurrent unit-based RNN (GRU-RNN) were found to be very suitable for dose-volume histogram (DVH) prediction in our previously published work. Using the dosimetric information generated by nonmodulated beams of different orientations, the GRU-RNN model was capable of accurate DVH prediction for nasopharyngeal carcinoma (NPC) treatment planning. On the basis of our previous work, we proposed an improved approach and aimed to further improve the DVH prediction accuracy as well as study the feasibility of applying the proposed method to relatively small-size patient data.

**Methods:**

Eighty NPC volumetric modulated arc therapy (VMAT) plans with local IRB's approval in recent two years were retrospectively and randomly selected in this study. All these original plans were created using the Eclipse treatment planning system (V13.5, Varian Medical Systems, USA) with ≥95% of PGTVnx receiving the prescribed doses of 70 Gy, ≥95% of PGTVnd receiving 66 Gy, and ≥95% of PTV receiving 60 Gy. Among them, fifty plans were used to train the DVH prediction model, and the remaining were used for testing. On the basis of our previously published work, we simplified the 3-layer GRU-RNN model to a single-layer model and further trained every organ at risk (OAR) separately with an OAR-specific equivalent uniform dose- (EUD-) based loss function.

**Results:**

The results of linear least squares regression obtained by the new proposed method showed the excellent agreements between the predictions and the original plans with the correlation coefficient *r* = 0.976 and 0.968 for EUD results and maximum dose results, respectively, and the coefficient *r* of our previously published method was 0.957 and 0.946, respectively. The Wilcoxon signed-rank test results between the proposed and the previous work showed that the proposed method could significantly improve the EUD prediction accuracy for the brainstem, spinal cord, and temporal lobes with a *p* value < 0.01.

**Conclusions:**

The accuracy of DVH prediction achieved in different OARs showed the great improvements compared to the previous works, and more importantly, the effectiveness and robustness showed by the simplified GRU-RNN trained from relatively small-size DVH samples, fully demonstrated the feasibility of applying the proposed method to small-size patient data. Excellent agreements in both EUD results and maximum dose results between the predictions and original plans indicated the application prospect in a physically and biologically related (or a mixture of both) model for treatment planning.

## 1. Introduction

Due to the complex tumor volumes in close proximity to critical structures, the nasopharyngeal carcinoma (NPC) radiation therapy (RT) plan was of great difficulty and experience-dependent [1–4]. In recent years, numbers of researches to aid in treatment planning using knowledge-based planning (KBP) techniques had improved the consistency of the plan quality and reduced the required optimization time [5–13]. The most popular tools [14–18] were developed to predict the dose-volume histogram from the organ at risk (OAR)—planning target volume (PTV) anatomy, which could assist in treatment planning by giving the appropriate OAR constraints and enabling the production of high-quality plans. The most widely used tools for quantifying the OAR-PTV anatomy, namely, the overlap volume histogram (OVH) [15, 16] and the distance-to-target histogram (DTH) [17, 18], were equivalent when the Euclidean form of the distance function was used in the DTH.

Compared to 3D-dose prediction [19–32] in the stage of academic research, DVH prediction has been clinically applied for years; for example, the commercial software named RapidPlan was developed based on the DTH approach by Varian Medical Systems (Palo Alto, California, US). However, one concern regarding the DTH and OVH was that their simplicity might lead to inaccurate presentation of the interpatient variations in anatomical features, which might have an impact on the dose deposition [12, 15, 33]. Another concern regarding the existent research was that the ignorance of the radiobiological difference in different structures or the different key features make dose distribution acceptable or unacceptable in clinic. For example, for an organ like the spinal cord, the maximum dose was considered to have the highest priority.

In our previously published works [12, 13], a multilayer gated recurrent unit-based recurrent neural network (GRU-RNN) was established to predict the DVHs for NPC treatment planning using the DVHs generated by the nonmodulated beams of different orientation. Using dosimetric information such as GRU-RNN inputs, the GRU-RNN was capable of accurate DVH prediction. Similar results were also obtained by other dosimetric information-driven researches [11, 30, 31]. RNN and its variants, such as the GRU-RNN used in this study, were particularly suitable for predicting the entire DVH. Its directionality was of great relevance for predicting the sequential data, such as DVH, a monotone decreasing sequence. And more importantly, compared to other models such as CNN, a great reduction of the parameter number in RNN and its variants indicated great potential in robust learning when applying to small-size data. The equivalent uniform dose (EUD) was the homogeneous dose inside an organ that has the same radiobiological effect as the given arbitrary dose distribution [34]. On the basis of our previous work, an EUD-based loss function was introduced in this study. By considering biological characters in different structures, the new method could pay more attention to the key dosimetric features such as maximum dose for the spinal cord and make the predicted DVH of more clinical value.

Aiming to improve the DVH prediction accuracy for NPC RT treatment planning, we proposed an improved approach in this work, which trained every OAR separately using a simplified GRU-RNN model with an equivalent uniform dose- (EUD-) based loss function, and study the feasibility of applying the proposed method to relatively small-size patient data.

## 2. Materials and Method

### 2.1. Data Acquisition

**Figure 1:**
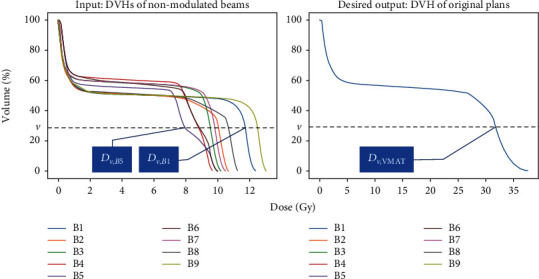
An example of DVHs generated by nonmodulated beams, *B*1, *B*2, *B*3, ⋯, *B*9, with a gantry angle of 160, 200, 240, 280, 320, 0, 40, 80, and 120 degrees and DVH of an original VMAT plan from a patient spinal cord.

80 NPC volumetric modulated arc therapy (VMAT) plans in recent two years with local IRB's approval were retrospectively and randomly selected for this study. Of these original plans, 50 were randomly selected for training and the remaining were used for testing. Following the ICRU-83 report, radiation oncologists delineated the gross tumor volume of the nasopharynx (GTVnx), the gross tumor volume of the metastatic lymph node (GTVnd), the clinical target volume (CTV), and the OARs in the planning CT. A margin of 3 mm was applied around the GTVnx, GTVnd, and CTV to create the planning GTVnx (PGTVnx), the planning GTVnd (PGTVnd), and the planning CTV (PTV), respectively. All the original VMAT plans were created using the Eclipse treatment planning system (V13.5, Varian Medical Systems, USA) with ≥95% of PGTVnx receiving the prescribed doses of 70 Gy, ≥95% of PGTVnd receiving 68 Gy, and ≥95% of PTV receiving 60 Gy. In this work, the DVHs were resampled by volume bin in percentage (1% in practice) rather than in absolute volume or dose values, making the DVHs of equal length. The DVHs of the nonmodulated beams were generated by a nine-field conformal plan with multileaf collimators fitting to PTV and normalizing 95% of PGTV dose to 70 Gy. An example of DVHs induced by nonmodulated beams and that of the original plan from a patient's spinal cord are shown in Figure 1.

### 2.2. GRU-RNN

**Figure 2:**
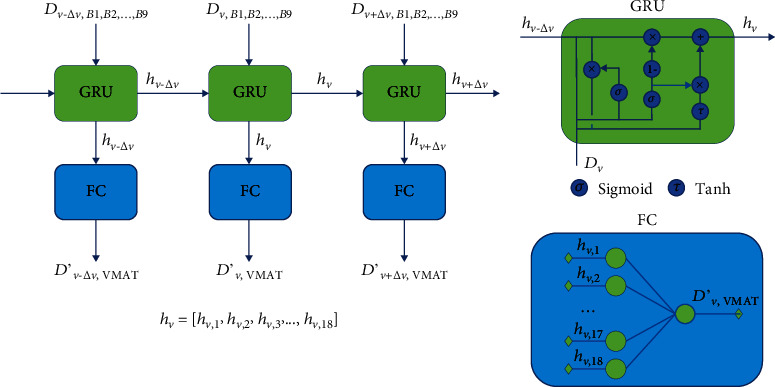
Architecture of the GRU-RNN model. In the practical experiments, Δ*v* was 1% OAR volume.

A single-layer GRU-RNN as shown in Figure 2 was established using the PyTorch (Facebook, US) framework for DVH prediction. *D*_*v*_ was the dose of original plans at percent volume *v* as shown in Figure 1, *D*′_*v*_ was the predicted dose, and *h*_*v*_ was the hidden state at volume *v*. A dropout layer was inserted between GRU and FC to randomly zero the parameters of *h*_*v*_ with a probability of 0.5. The GRU was trained by the Adam optimizer with the goal of minimizing the loss function by a learning rate of 1*e* − 3.

### 2.3. Loss Function

The concept of equivalent uniform dose (EUD) assumes that any two dose distributions are equivalent if they cause the same radiobiological effect, which can be calculated as follows [34]:

(1) $$\begin{array}{c} \text{EUD}={\left(\underset{i=0}{\sum }v\cdot D_{i}^{a}\right)}^{1/a}. \end{array}$$

Equation (2) shows that different dose values take different weights in EUD calculation when *a* ≠ 1.

(2) $$\begin{array}{c} \frac{d\left(\text{EUD}\right)}{d\left(D_{i}\right)}={\left(\text{EUD}\right)}^{1-a}*D_{i}^{a-1}, \end{array}$$

(3) $$\begin{array}{c} s\left(D_{i},a\right)=\frac{D_{i}^{a-1}}{\sum_{i=0}D_{i}^{a-1}}. \end{array}$$

*s*(*a*) represents the sensitivity of the dose value to the EUD value. The loss function to be minimized in this study is defined as equation (4) to meet the different dose requirements for different OARs. For example, for an OAR like the spinal cord, the maximum dose is considered to have the highest priority; therefore, the GRU-RNN model was individually trained with *k* ≫ 1.

(4) $$\begin{array}{c} f\left(\text{DV}{\text{H}}^{\prime },\text{DVH},k\right)=\frac{1}{n}\overset{n}{\underset{p}{\sum }}\underset{i=0}{\sum }s\left(D_{i},k\right){\left(D_{i}^{\prime }-D_{i}\right)}^{2}. \end{array}$$

**Figure 3:**
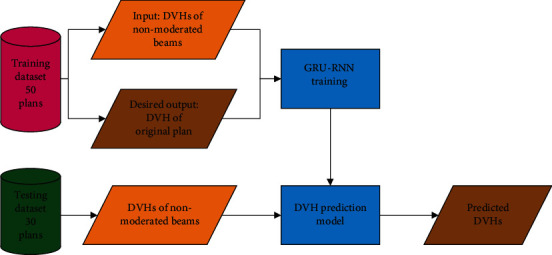
The flowchart showed the dosimetric information of nonmodulated beam-driven DVH prediction.

Here, DVH and DVH′ were the DVH of the original plan and prediction. *k* was a positive integer and determined by trial and error with the goal of accurately predicting both EUD and maximum dose (only for serial OARs). The trial and error results were shown, when 8 was used for the brainstem, 15 for the spinal cord, 3 and 2 for the left and right optic nerves, respectively, and 1 for the chiasm, larynx, parotid glands, and temporal lobes; the most accurate EUD results (recommended by Allen Li et al. [35], *a* = 8 was used for serial organs including the brainstem, spinal cord, optic nerves, and chiasm and *a* = 1 was used for parallel organs including the parotids, larynx, and temporal lobes in EUD calculation) were obtained. A flowchart of the dosimetric information of nonmodulated beam-driven DVH prediction is shown in Figure 3.

### 2.4. Model Evaluation

*μ* ± *σ* was calculated to evaluate the GRU-RNN performance:

(5) $$\begin{array}{c} \delta_{i}=D_{i}^{\prime }-D_{i}, \end{array}$$

(6) $$\begin{array}{c} \mu =\frac{1}{n}\overset{n}{\underset{i=1}{\sum }}\delta_{i}, \end{array}$$

(7) $$\begin{array}{c} \sigma =\sqrt{\frac{\sum {\left(\delta_{i}-\mu \right)}^{2}}{n}}, \end{array}$$

where *i* represents a testing patient and *n* is the number of testing patients, *D*_*i*_′ denotes the predicted EUD or maximum dose, and *D*_*i*_ denotes the result of the original plan. The results were also compared to those obtained by our previous work [13] to demonstrate the improvements of the new proposed method in this study. Wilcoxon signed-rank tests were employed to compare the prediction error, *δ*_*i*=1,2,⋯,30_ , among the 30 testing patients between the proposed method and the previous one. Differences were considered statistically significant at *p* < 0.05.

## 3. Results

**Figure 4:**
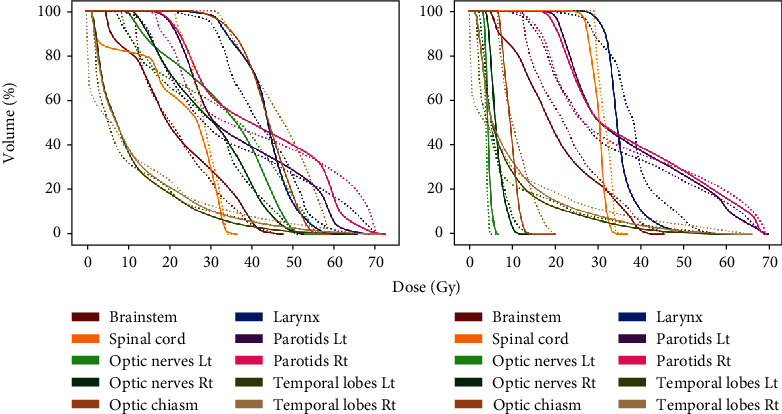
Comparisons between DVHs of predictions and original plans from two testing patients. Dash line: predicted DVHs; solid line: original plans' DVHs; Lt: left side; Rt: right side.

Two randomly selected testing patients' DVH prediction results are demonstrated in Figure 4. Though obvious differences could be seen in the deposited dose between two patients especially at the optic nerves and chiasm, the predicted DVHs of the two patients were still very close to the original plans.

**Figure 5:**
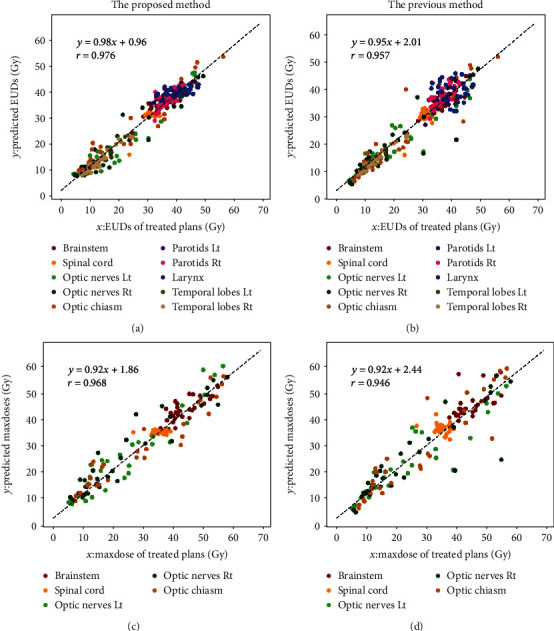
The correlations of the EUD (a, c) and maximum dose (b, d) results between the predictions and the original plans for the 30 test patients.

The correlations of the EUD and maximum dose results between the predictions and the original plans for the 30 test patients are plotted in Figure 5. The results of linear least squares regression showed that the predicted EUD results and maximum dose results of the proposed method in this study were both in good agreement with the original plans with correlation coefficient *r* = 0.976 (Figure 5(a)) and 0.968 (Figure 5(c)), respectively. The prediction results obtained following our previous work [13] are also demonstrated in Figures 5(b) and 5(d), and the coefficient *r* values were 0.957 (Figure 5(a)) and 0.946 (Figure 5(c)). The proposed method in this study has better consistency between the predicted results and those of original plans, which could be seen in the scatter plots and the coefficient *r* results quantitatively.

Table 1 provides a summary (*μ* ± *σ*) and a comparison (*p* value) over the prediction error (*δ*_*i*=1,2,⋯,30_) of the EUD results and maximum dose results in the 30 testing patients. The *μ* ± *σ* results showed *σ* decreased by the proposed method in almost all the OARs except for temporal lobes for both maximum doses and EUDs. The patient-wise Wilcoxon signed-rank tests results over *δ*_*i*_ between the proposed and the previous method [12] showed that the proposed method could significantly improve the EUD prediction accuracy of the brainstem, spinal cord, and temporal lobes with *p* value < 0.01.

**Figure 6:**
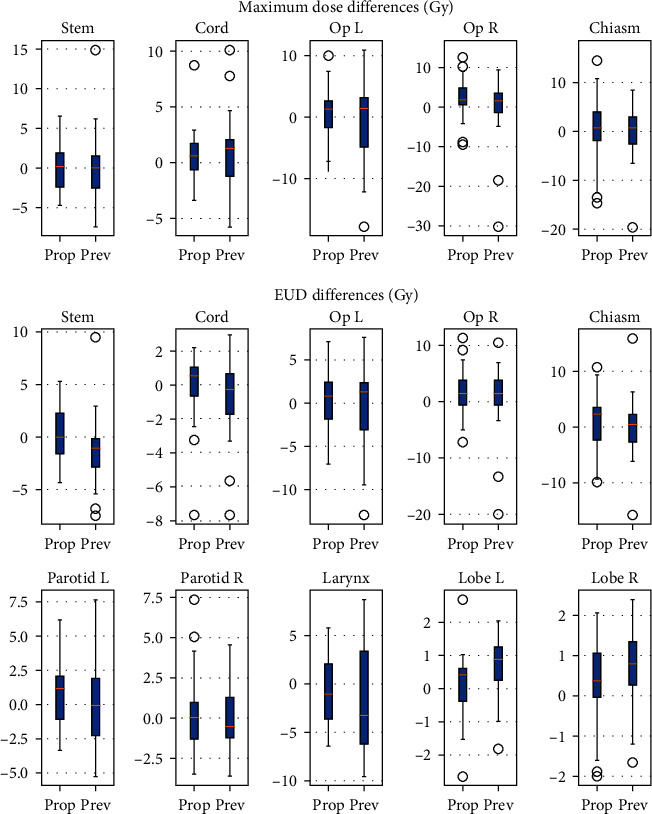
The differences between results of treated plans and the predicted results obtained by the proposed method (Prop) as well as the previous method (Prev) for the brainstem (stem), spinal cord (cord), left and right optic nerves (op L and op R), optic chiasm (chiasm), left and right parotid glands (parotid L and parotid R), larynx, and left and right temporal lobes (lobe L and lobe R).

The differences between results of the original plans and the predictions obtained by the proposed method (Prop) as well as the previous method (Prev) were expressed with a boxplot in Figure 6. The bottom and top of each box were the 25th and 75th percentiles of the differences, respectively. The distance between the bottom and top of each box was the interquartile difference range, and the lines in the middle of each box were the median differences. The whiskers were lines extending above and below each box. Whiskers went from the end of the interquartile range to the largest difference. Differences beyond the whisker length were marked as outliers, which were more than 1.5 times the interquartile range away from the bottom or top of the box. The proposed method in this study as shown in Figure 6, compared to the previous method in both maximum doses and EUDs, had the median differences closer to 0, smaller interquartile differences, and less outliers indicating better prediction accuracy and better reliability.

## 4. Discussion

In this study, we proposed an improved approach, which trained every OAR separately with a simplified GRU-RNN model and an equivalent uniform dose- (EUD-) based loss function. As shown in Figure 5, the new proposed method in this study improved the consistency of EUD results and maximum dose results between the predictions and original plans. For parallel OARs such as the larynx, parotid glands, and temporal lobes, the *k* values in equation (4) were set to 1.0, making the *s*(*D*_*i*_, *k*) term ineffective. The improved prediction accuracy in these OARs indicated that training different OARs separately was helpful. In our preliminary trials, we had also trained the GRU-RNN with *k* = 1 for the brainstem and spinal cord, the EUD results of *μ* ± *σ* were 0.76 ± 2.94 Gy and 0.29 ± 2.03 Gy, and the maximum dose results of *μ* ± *σ* were 0.58 ± 3.16 Gy and 0.69 ± 2.37 Gy. The results showed the *s*(*D*_*i*_, *k*) term was able to improve the prediction accuracy of EUD and maximum dose. Excellent agreements in both EUD values and maximum doses between the predictions and original plans obtained by the new proposed method indicated the application prospect in a physically and biologically related (or a mixture of both) model for treatment planning.

The GRU-RNN models were trained from only 50 DVH samples, which could reasonably be considered relatively small-size data. The excellent agreements of the results between the predictions and original plans fully demonstrated the feasibility of applying the proposed method to small-size patient data. As mentioned above, RNN and its variants, such as GRU-RNN in this study, are particularly suitable for predicting the entire DVH rather than only fixed amount of interesting points. Compared to CNN and other models, its directionality was of great relevance for predicting the sequential data, such as DVH, a monotone decreasing sequence. In this study, we trained different OARs separately and focused the training attention on the interpatient variations in deposited dose with no need of figuring out the different OARs. Decreasing the training difficulty allowed the usage of a further simplified model, a single-layer GRU-RNN in this study, which was of great significance in small-size sample training. In addition, due to the greatly reduced complexity of the modeling task, the training time is less than 100 seconds for every OAR with a computer equipped with i7-4770K CPU, Geforce GTX Titan GPU, and 16 GB memory.

Different *k* values of 3.0 and 1.6 for optic nerves seem unreasonable and illogical due to the similar size, the symmetrical distribution, and the same biological characteristics in left\right optic nerves. A possible reason might be that the small size of the samples was not enough to represent the broader cases. In other words, the proposed method in this study might be a possible way to backtrack the value of “*a*” in equation (1) with the results of the existing plan data, but the values in this study seem too data-dependent to be repeated.

## 5. Conclusion

The accuracy of DVH prediction achieved in different OARs showed the great improvements compared to the previous works [12, 13] and the potential of this approach being extended to other disease sites. More importantly, the effectiveness and robustness showed by the simplified and well-trained GRU-RNN models trained from relatively small-size DVH samples fully demonstrated the feasibility of applying the proposed method to small-size patient data. In addition, excellent agreements in both EUD values and maximum doses between the predictions and original plans indicated the application prospect in a physical and biologically related (or a mixture of both) model for treatment planning.

## Figures and Tables

**Table 1:** A summary (*μ* ± *σ* over *δ*) and a comparison (patient-wise *p* value over *δ*) of prediction accuracy in maximum doses and EUDs for the 30 test patients. Prop was the results obtained by the proposed method, and Prev was the results obtained by the previous method.

OARs	*δ*	*μ* ± *σ* (Gy)		*p* value
		Prop	Prev	Prop vs. Prev
Brainstem	*D*_max_	0.34 ± 3.22	−0.27 ± 4.23	0.16
	EUD	0.64 ± 2.61	−1.24 ± 3.11	<0.01
Spinal cord	*D*_max_	0.58 ± 2.29	0.79 ± 3.23	0.29
	EUD	0.10 ± 1.96	−0.62 ± 2.20	<0.01
Optic chiasm	*D*_max_	−0.33 ± 6.18	−0.50 ± 6.02	0.57
	EUD	0.089 ± 4.58	0.27 ± 5.27	0.51
Optic nerves Lt	*D*_max_	0.68 ± 4.13	−0.52 ± 6.11	0.27
	EUD	0.50 ± 3.13	−0.32 ± 4.65	0.43
Optic nerves Rt	*D*_max_	1.23 ± 4.70	0.02 ± 7.46	0.37
	EUD	1.22 ± 3.71	0.26 ± 5.20	0.41
Larynx	EUD	−0.66 ± 3.46	−1.48 ± 5.59	0.22
Parotids Lt	EUD	0.64 ± 2.32	0.12 ± 2.80	0.13
Parotids Rt	EUD	0.24 ± 2.47	0.07 ± 2.14	0.37
Temporal lobes Lt	EUD	0.17 ± 0.92	0.69 ± 0.84	<0.01
Temporal lobes Rt	EUD	0.32 ± 1.07	0.73 ± 0.98	<0.01

## Data Availability

All datasets generated for this study are included in the article.
